# Juxtacortical Mandibular Chondrosarcoma during pregnancy: A case report

**DOI:** 10.4317/jced.53630

**Published:** 2017-05-01

**Authors:** Paolo Cariati, Almudena Cabello-Serrano, Fernando Monsalve-Iglesias, Miguel Perez-de Perceval-Tara, Ildefonso Martinez-Lara

**Affiliations:** 1 Oral and Maxillofacial surgery resident. Hospital Universitario Virgen de las nieves, Granada, Spain; 2 Maxillofacial Surgeon. Hospital Universitario Virgen de las nieves, Granada, Spain

## Abstract

Chondrosarcoma is one of the most common malignant bone tumors in adults. It use to affect upper arm, pelvis and thigh bone. A wide surgical extirpation represent the gold standard to treat this disorder. In fact, radiotherapy and chemotherapy are no useful. Interestingly, chondrosarcoma is rare in head and neck (HNCS) and extremely uncommon during pregnancy. Thus, there is a lack of evidence about the proper treatment in these cases. A wide surgical extirpation is also considered the most effective procedure in HNCS. There are no consistent evidences about the he role of radiation and chemotherapy. In view of that, the present study describes a case of juxtacortical mandibular chondrosarcoma affecting a 28-year-old pregnant woman. After a multidisciplinary analysis of the case, we decided to treat the patient with a wide surgical resection and and immediately reconstruction.

** Key words:**Mandibular chondrosarcoma, pregnancy, surgical extirpation, radiotherapy, chemoteraphy.

## Introduction

Chondrosarcoma is a malignant cartilaginous tumor characterized by the formation of cartilage, but not of bone, by tumor cells (1). The most common location of this tumor are pelvis, extremities and ribs. The incidence of involvement of head and neck sites varies from 5% to 12% (2). Larynx, thyroid cartilage, and arytenoids are the most common localizations of injurie. Moreover, chondrosarcomas could affect other areas of the craniofacial area such as the mandible, maxilla, nose, paranasal sinuses, base of the skull and nasopharynx. Nevertheless, chondrosarcoma is rare in head and neck. This malignant neoplasm cause only 0.1% of all head and neck neoplasms. In the head and neck, usually begins between the fourth and the seventh decades of life (3). The etiology appear to be related with the uncontrolled proliferation of Merkel Cells (embryonic remains).

In this line, chondrosarcoma is also uncommon during pregnancy. In fact, only 10 cases of gestational chondrosarcomas was described in the literature during the last 25 years. Only one of these cases affected craniofacial area (4). The effect of pregnancy on growth features of chondrosarcomas remains unclear. Some studies suggest a possible growth-enhancing effect of altered hormone levels on various bone tumors.

Due to the rarity of head and neck chondrosarcoma (HNCS) there are no established treatment protocols. Many of the current treatment approaches stem from protocols developed and tested for chondrosarcomas of other more frequent locations. However, the adequate surgical resection remains the gold standard for the treatment of the jaw chondrosarcoma. In fact, the real role of chemotherapy and radiotherapy is still controversial (5).

## Case Report

The article describes a case of a juxtacortical chondrosarcoma of the mandible affecting a 28-year-old woman. Interestingly, she was 31 weeks pregnant.

**Figure 1:**
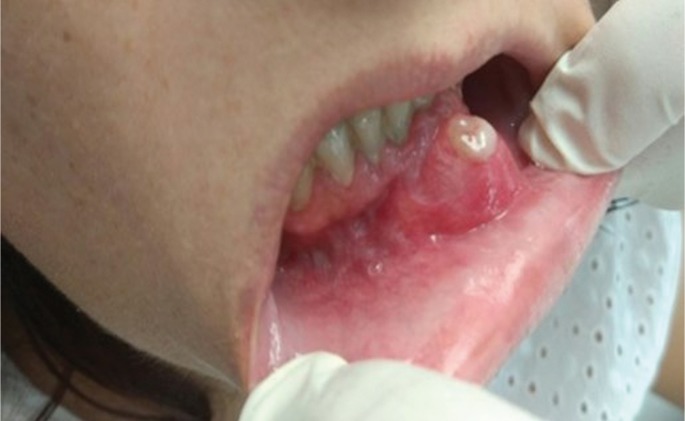
Clinical image of mandibular chondrosarcoma. Patient referred a rapid growth of the injury. She noted a small lump three or four weeks before. The aggressiveness of the lesion is evident.

**Figure 2:**
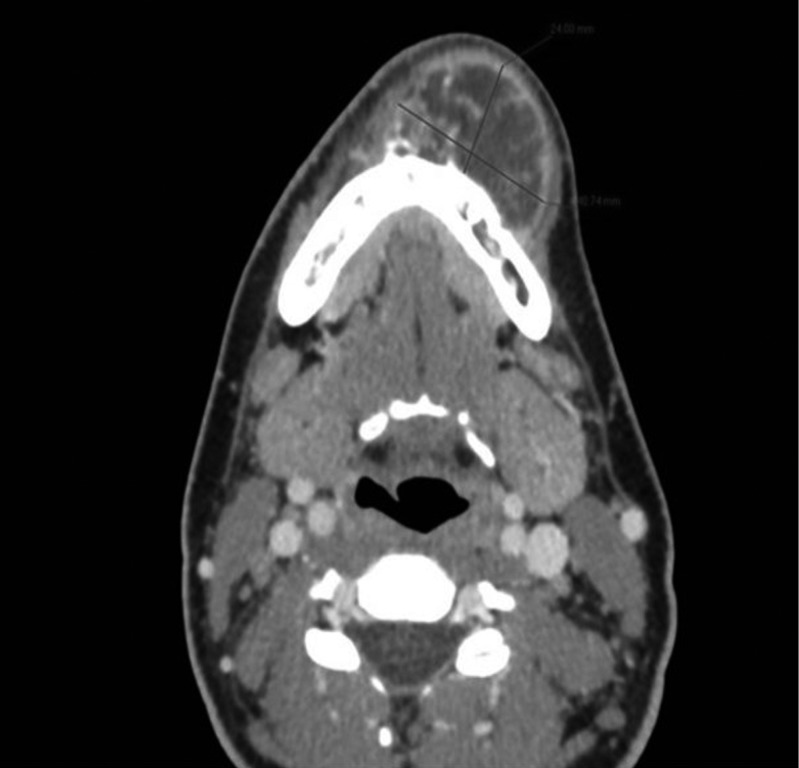
Sagittal CT images of mandibular chondrosarcoma.

More in detail, the patient referred the onset of a painless lump in the mandibular symphysis at the emergency department of our hospital. The lesion appeared spontaneously a few weeks earlier. No trismus, paresthesia or other symptoms were reported by the patient. At the exploration, the mass was fixed to the jaw without mobility. Moreover, the patient related a rapid growth (Fig. 1). Against this backdrop, the emergency physician alerted the maxillofacial surgeon on call. Consequently, the patient was referred to maxillofacial outpatient clinic. Once there, we decided to carry out a diagnostic biopsy. This test evidenced the presence of chondrosarcoma cells in the specimen. Therefore, after an accurate analysis of the case the diagnosis of juxtacortical mandibular chondrosarcoma was obtained. Considering that patient was almost 8 months pregnant at the time, a non-contrast CT scan of cervicofacial area was performed when the biopsy result were obtained. Labor was induced at 36 weeks of pregnancy. One week later, we completed the diagnostic process with a contrast CT scan. Mandibular bone was extensively affected by the tumor. Jaw was involved from 43 to 36 (Fig. 2).

**Figure 3:**
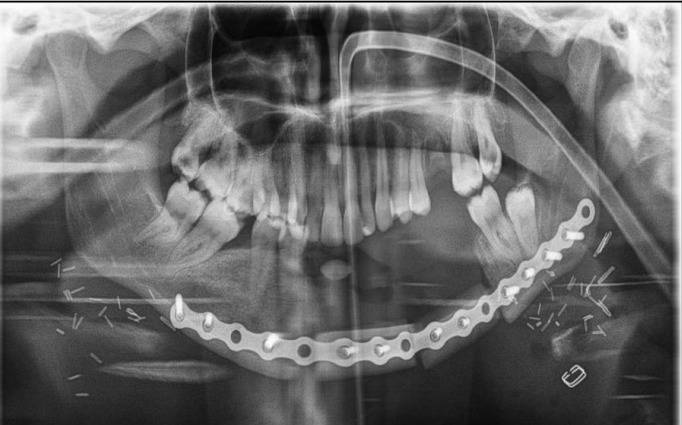
Post-op panoramic x-ray of jaw reconstruction.

Thus, after the presentation of all the evidence we classified the cancer as cT3N0M0. In the oncological committee of our hospi-tal, we decided to treat the case with a wide surgical resection and inmediately reconstruction. Surgery was performed three weeks after delivery. The extension of the injury obliged to perform a mandibulectomy from the tooth 44 to 37, including the skin of mental and submental region. The reconstruction was performed by using a microsurgical scapular flap (Fig. 3). Postoperative examination confirmed the diagnosis of juxtacortical HNCS moderately differentiated with resection margins free. The patient has no clinical signs of recurrence 18 months after surgery.

## Discussion

The incidence of CHS in the mandible is rare. Only 30 cases have been reported to date (MEDLINE 1966-2016). The clinical behavior is variable. However, the majority (80%-90%) are low-grade malignant tumors. The median age of presentation varies between 17 to 75 years and it occurs more often in females (6). Whereas, chondrosarcoma is more common in men if all cervico-facial area is considered (HNCS). According to the literature, the time required to make a diagnosis varies generally between 6 and 24 months. This delay might be due to confusion with other inflammatory pathologies (7). However, in our case diagnostic was very quick (approximatively 1 month after first symptom). In fact, the tumor showed a rapid and progressive growth. This might be related to hormonal shifts caused by pregnancy. There is only one documented case of HNCS during pregnancy (8). Hence, there is insufficient evidence to characterize this pathology.

Panoramic x-ray and CT or magnetic resonance imaging are essentials for a proper diagnosis and analysis of the tumor extension. Additionally, an incisional biopsy is needed to complete the diagnosis. In this line, an aspiration biopsy is not recommended.

A number of other lesions should be considered in the differential diagnosis such as Epulis, Haemangioma and fibrosarcoma (7).

Wide surgical resection is currently the gold standard to treat HNCS. Resection must be wide (> 2-3 cm). Close or positive margins are related with an increased risk of recurrence. Local recurrence is the main cause of death. Other factors that influence the global survival are represented by stage at diagnosis, histopathological grade and location. In general, lymph node metastases and distant metastases are infrequent. Survival rates for HNCS range between 44 and 88 % (9).

Postoperative radiotherapy and systematic chemotherapy might be useful to improve disease free survival and overall survival. Notwithstanding, there are no consistent evidences about the effectiveness of these treatment. Chondrosarcoma appears to be resistant to radiation treatment. Postoperative radiotherapy is recommended only for high-grade tumors and for unresectable lesions. On the other hand, an effective chemotherapy regimen does not exist at the moment (10).

A wide surgical resection is also the first choice of treatment in skeletal chondrosarcoma of during pregnancy (4). In our case, we treat the patient with surgical resection and immediately reconstruction. Considering the wide surgical margins and lack of evi-dence about the effectiveness of radiotherapy and chemotherapy we decided to treat the patient only with surgery. Moreover, these treatment are not very effectively in skeletal chondrosarcoma. No signs of recurrence or metastases were evidenced 18 months after surgery. Despite this a close follow up is mandatory. Tumor might provoke metastases several years after diagnosis.

Resuming, this case stresses the importance of wide surgical resection. However, no evidences exist and more research is needed in this field. This particularly applies to HNCS during pregnancy.
